# Risk factors of radiation-induced acute esophagitis in non-small cell lung cancer patients treated with concomitant chemoradiotherapy

**DOI:** 10.1186/1748-717X-9-54

**Published:** 2014-02-15

**Authors:** ZiCheng Zhang, Jin Xu, Tao Zhou, Yan Yi, HongSheng Li, HongFu Sun, Wei Huang, DongQing Wang, BaoSheng Li, GuoGuang Ying

**Affiliations:** 1 Tianjin Medical University Cancer Institute and Hospital, Tianjin, China; 2 Department of Radiation Oncology, Shandong Cancer Hospital and Institute, Jinan, China; 3 Tianjin Key Laboratory of Cancer Prevention and Therapy, Tianjin, China; 4 Tianjin Medical University, Tianjin, China

**Keywords:** Acute esophagitis, Concomitant chemoradiotherapy, Risk factors of acute esophagitis, Non-small-cell lung cancer

## Abstract

**Background:**

To analyze the clinical and dosimetric risk factors of acute esophagitis (AE) in non-small-cell lung cancer (NSCLC) patients treated with concomitant chemoradiotherapy.

**Methods:**

Seventy-six NSCLC patients treated with concomitant chemoradiotherapy were retrospectively analyzed. Forty-one patients received concomitant chemoradiotherapy with vinorelbine/cisplatin (VC), 35 with docetaxel/cisplatin (DC). AE was graded according to criteria of the Radiation Therapy Oncology Group (RTOG). The following clinical and dosimetric parameters were analyzed: gender, age, clinical stage, Karnofsky performance status (KPS), pretreatment weight loss, concomitant chemotherapy agents (CCA) (VC vs. DC), percentage of esophagus volume treated to ≥20 (V20), ≥30 (V30), ≥40 (V40), ≥50 (V50) and ≥60 Gy (V60), and the maximum (Dmax) and mean doses (Dmean) delivered to esophagus. Univariate and multivariate logistic regression analysis were used to test the association between the different factors and AE.

**Results:**

Seventy patients developed AE (Grade 1, 19 patients; Grade 2, 36 patients; and Grade 3, 15 patients). By multivariate logistic regression analysis, V40 was the only statistically significant factor associated with Grade ≥2 AE (p<0.001, OR = 1.159). A V40 of <23% had a 33.3% (10/30) risk of Grade ≥2 AE, which increased to 89.1% (41/46) with a V40 of ≥23% (p<0.001). CCA (p =0.01; OR = 9.686) and V50 (p<0.001; OR = 1.122) were most significantly correlated with grade 3 AE. A V50 of <26.5% had a 6.7% (3/45) risk of Grade 3 AE, which increased to 38.7% (12/31) with a V50 of ≥26.5% (p = 0.001). On the linear regression analysis, V50 and CCA were significant independent factors affecting AE duration. Patients who received concomitant chemotherapy with VC had a decreased risk of grade 3 AE and shorter duration compared with DC.

**Conclusions:**

Concomitant chemotherapy agents have potential influence on AE. Concomitant chemotherapy with VC led to lower risk of AE compared with that using DC. V40 and V50 of esophagus can predict grade ≥2 and ≥3 AE, respectively.

## Background

Lung cancer is currently the most common malignancy and also the leading cause of cancer-related mortality in the world [1]. Non-small cell lung cancer (NSCLC) accounts for approximately 85% of all lung cancers [2]. About one third of patients with NSCLC present with locally advanced disease at the time of diagnosis [3]. The recommended treatment of patients presenting with inoperable stage-III NSCLC and a good performance score is concomitant chemoradiotherapy [4]. But the use of concurrent chemoradiation increases the risk of esophagitis, which remains the primary radiation-related toxicity [5]. At present, a two-drug regimen combining a platinum agent with either vinorelbine or docetaxel is considered as the standard practice of chemoradiotherapy for local advanced or advanced NSCLC. Whether the severity of AE is influenced by concomitant chemotherapy agent (CCA) is uncertain. The purpose of this work was to perform prospective study to determine risk factors for AE in NSCLC patients treated with radiation combined with VC or DC concomitant chemotherapy.

## Methods

### Patients

Between October 2006 and March 2009, 76 patients diagnosed with NSCLC were enrolled in this study. All patients had histologically or cytological proven NSCLC (40 patients had squamous-cell carcinoma, 32 adenocarcinoma, 1 adenosquamous carcinoma, 1 large cell carcinoma, 2 others). There were 59 men and 17 women. The median age was 59 years (range, 33–77 years). All patients were staged according to the AJCC 2002 classification. Three patients had stage II, 32 stage IIIa, 37 stage IIIb, and 4 stage IV. Of these patients, 11 patients had undergone surgical intervention before chemoradiotherapy.

### Chemotherapy

All patients received concomitant chemoradiotherapy, which were administered by medical oncologists according to our institutional standards. Of these patients, 41 received concomitant chemotherapy with VC, 35 with DC. Concomitant chemotherapy regimens were summarized in the Table 1. Seventy-three patients had been treated with concomitant chemotherapy starting on the first day of radiation therapy, 3 on the second day. Twelve patients had received induction chemotherapy. After completion of radiotherapy, 71 patients received consolidation chemotherapy in our institution, and 5 patients were followed up.

**Table 1:** **Concomitant chemotherapy regimens (*n*** **= 76)**

**Concomitant chemotherapy regimens**	**Cycles**	**Patients number ( *n *)**
Vinorelbine (20 mg/m^2^, days 1, 8) and cisplatin (25 mg/m^2^, day 1,2,3), every 21 days.	2	5
Vinorelbine (25 mg/m^2^, days 1, 8) and cisplatin (25 mg/m^2^, day 1,2,3), every 21 days.	2	36
Docetaxel (30 mg/m^2^, days 1, 8) and cisplatin (25 mg/m^2^, day 1,2,3), every 21 days.	2	6
Docetaxel (35 mg/m^2^, days 1, 8) and cisplatin (25 mg/m^2^, day 1,2,3), every 21 days.	2	28
Docetaxel (40 mg/m^2^, days 1,8) and cisplatin (25 mg/m^2^, day 1,2,3), every 21 days.	2	1

### Radiotherapy treatment and dosimetric parameters

Seventy-one patients were treated with three-dimensional conformal radiotherapy (3D-CRT), 5 with intensity modulated radiation therapy (IMRT). Patients were set up in treatment position and immobilized using vacuum bag to improve the reproducibility during daily treatments. Computed Tomography (CT) scans with a slice thickness of 5 mm thick was obtained from the lower end of the cricoid cartilage to the lower edge of the liver. The obtained CT images were directly transmitted to a 3D planning system (Pinnacle3, Philips Medical Systems). Gross tumor volume (GTV) was delineated to encompass all detectable tumors, including primary mass and metastatic regional lymph nodes observed on CT. Planning target volume (PTV) included GTV and margins of 5 to 12 mm for lymph nodes, 8 to 20 mm for primary tumor. Fifty-eight patients (76.3%) were treated with involved-field irradiation, 18 (23.7%) with elective nodal irradiation. The external surface of the esophagus was contoured uniformly on each of the axial images of the planning CT by the same physician. The normal esophagus was defined from the inferior border of the cricoid cartilage to the gastroesophagea junction. All patients were treated with conventional fractionation scheme, 5 days per week, with fractional dose of 2.0 Gy for 70 patients and 1.8 Gy for 6 patients. The median of fraction number was 30, ranging 28–35. At least 90% of a PTV was required to be covered by the prescription dose. Dose constraints for normal tissue used in the planning process were as follows: V_20_ < 33% (i.e., ≤ 33% of total lung volume received ≥ 20 Gy), and spinal cord D_max_ < 48 Gy. No dose limits were used for the esophagus, trachea, and heart. Dose distribution was calculated with tissue heterogeneity correction. A series of dose volume parameters including Dmean, Dmax, V20, V30, V40, V50 and V60 were recorded for normal structures. Radiotherapy was delivered with linear accelerators using 6 or 15-MV X-rays. Treatment techniques typically included initial anterior-posterior beams followed by oblique off-cord beams. Most of plans employed 4 to 6 fields. Respiration motion was managed by using the Active Breathing Coordinator device (Elekta Inc.) for 18 patients (23.7%). Electronic portal images acquired with large orthogonal fields (18 cm × 18 cm) with 18 monitor units were used to guide daily patient positioning for 32 patients (42.1%), while cone-beam CTs were used to position patient at the first three treatments and once a week subsequently for 44 patients (57.9%).

### Clinical evaluation and follow-up

All patients were hospitalized during the course of radiotherapy, and were seen everyday as routine evaluation of the treatment. After completion of therapy, patients were followed up every month for the first 3 months and every 3 or 4 months thereafter. If necessary, telephone follow-up was conducted to acquire more information on radiation induced toxicity. AE (measured within 3 months of initiating radiotherapy) were evaluated according to the RTOG and European Organization for Research and Treatment of Cancer toxicity grading scale [6]. These data, including complaints of patients, the maximum toxicity grade, the first day of symptoms appeared and the first day of symptoms disappeared were recorded. Duration of AE was the gap between the first day of symptoms disappeared and the first day of symptoms appeared.

### Statistical analysis

The maximal grade and the AE duration for each patient were used in the statistical analysis. A descriptive statistical analysis was performed and results are reported as mean ± Standard deviation (SD) for continuous variable. The correlations among dosimetric parameters were assessed with Pearson Correlation coefficient test. The difference of dosimetric parameters between VC group and DC group were evaluated by *t* test. Mann–Whitney *U* test was used to verify the difference of occurring time. Patients experiencing grade 2 or worse, grade 3 or worse, were counted as positive events. The following factors: gender (male vs. female), age (continuous), clinical stage (≤IIIa vs. ≥IIIb), KPS (<90 vs. ≥90), pretreatment weight loss (<5% vs. ≥5%), CCA (VC vs. DC), Dmean, Dmax, V20, V30, V40, V50 and V60, were included in the statistical analysis. Univariate logistic regression analysis was used to test the association between the different factors and positive events (Grade ≥2 AE, Grade ≥3 AE). Pearson correlation analysis was used to correlate these parameters with duration (continuous). For multivariate analysis, the forward Wald procedure was performed using a logistic regression model containing all statistically significant variables in univariate analysis (p<0.10). Receiver operating characteristic (ROC) curves have been used to identify discriminate threshold. Linear regression model was used to evaluate their importance in duration (continuous). All statistical tests were 2-sided. Statistically significant differences are reported for p<0.05. Statistical analyses were performed using SPSS (version 15.0 for Windows).

## Results and discussion

### Results

Of all patients studied, fifteen patients experienced interruption in radiotherapy delivery, 5 due to AE (3 in DC group, 2 in VC group), 7 due to neutropenia, and 3 due to machine problems. For patients with radiotherapy interruptions, the median delay was 3 days (range, 1-5 days). After completion of radiotherapy, fifteen patients experienced delay of consolidation chemotherapy, 6 for neutropenia, 6 for AE (4 in DC group, 2 in VC group), 1 for transaminases, and 2 for refusal of patient. The median delay was 5 days (range, 1–13).

The mean ± SD of radiation dose to PTV was 60.7 ± 2.6 Gy (median 60Gy; range, 56–66 Gy). VC group had slightly numerically higher dosimetric parameters of esophagus than DC group, but there were no statistical difference by t test (p:0.293-0.616). Most of dosimetric parameters were closely correlated with one another when assessed by Pearson correlation coefficient test (r:0.439-0.980; p<0.001) except the correlation between Dmax and V20(r = 0.173, p = 0.136).

A total of 70 patients (92.1%, 70/76) developed grade ≥1 AE. Among them, 19 patients experienced grade 1 AE (25.0%, 19/76), 36 grade 2 (47.4%, 36/76) and 15 grade 3 (19.7%, 15/76). No grade 4 or 5 AE was observed. In the VC group, 5 patients experienced grade 0 (12.2%, 5/41), 11 grade 1 (26.8%, 11/41), 19 grade 2 (46.3%, 19/41) and 6 grade 3 (14.6%, 6/41). In the DC group, 1 patients experienced grade 0 (2.9%, 1/35), 8 grade 1 (22.9%, 8/35), 17 grade 2 (48.6%, 17/35) and 9 grade 3 (25.7%, 9/35).

### Predictors for grade ≥2 AE, grade ≥3 AE

Of the clinical factors investigated, such as gender, age, clinical stage, KPS, pretreatment weight loss and CCA, none was found to be significant for predicting grade ≥2 or grade 3 AE in univariate analysis (p<0.05). The CCA (VC vs. DC) was weakly related to grade 3 AE (p = 0.081, OR = 2.880). All dosimetric parameters investigated, including Dmax, Dmean, V20, V30, V40, V50 and V60 of esophagus, were found to be significantly associated with the risk of grade ≥2 or grade 3 AE (p<0.05) in univariate analysis. These results were summarized in Table 2. All dosimetric parameters were input into the multivariate logistic regression analysis. CCA was marginally significant and was also included in the multivariate analysis of grade 3 AE. V40 was found to be the only statistically significant factor associated with Grade ≥2 AE. CCA and V50 were most significantly correlated with grade 3 AE. Table 3 showed the result of multivariate analysis by SPSS15.0.

**Table 2:** Univariate analysis of dosimetric parameters associated with AE

**Variable**	**Grade ≥ 2 AE**		**Grade ≥ 3 AE**	
	***p***	***OR***	***p***	***OR***
Dmean (Gy)	<0.001	1.002	<0.001	1.001
Dmax (Gy)	0.001	1.001	0.007	1.001
V20 (%)	0.001	1.068	0.01	1.054
V30 (%)	<0.001	1.149	0.001	1.069
V40 (%)	<0.001	1.159	<0.001	1.087
V50 (%)	<0.001	1.138	<0.001	1.097
V60 (%)	<0.001	1.124	<0.001	1.094

*Abbreviations:AE* = acute esophagitis; *OR* = odds ratio; *Dmax* the maximum dose of the esophagus; *Dmean* = the mean dose of the esophagus; V20,30,40,50,60 = relative esophageal volume for radiation dose ≥20,30,40,50,60 Gy, respectively.

**Table 3:** Multivariate analysis of dosimetric parameters associated with AE

** Grade**	**Variable**	** B**	** S.E.**	**Wald**	**df**	** Sig.**	**Exp (B)**	**95.0% C.I. for EXP (B)**	
								**Lower**	**Upper**
Grade 2	V40	0.148	0.036	17.248	1	<0.001	1.159	1.081	1.243
	Constant	-2.760	0.811	11.574	1	0.001	0.063		
Grade 3	V50	0.115	0.030	15.204	1	<0.001	1.122	1.059	1.189
	CCA	2.271	0.886	6.565	1	0.010	9.686	1.705	55.016
	Constant	-8.389	2.138	15.393	1	<0.001	<0.001		

*Abbreviations*: *AE* acute esophagitis, *CCA* concomitant chemotherapy agents (vinorelbine/cisplatin vs. docetaxel/cisplatin), *V40,50* relative esophageal volume for radiation dose ≥40,50 Gy, respectively.

The predictive power of V40 for grade ≥2 AE was tested by ROC analysis. The areas under the ROC curve for grade ≥2 AE were 0.888 (95% CI: 0.813-0.963; p<0.001). These data had been used to identify discrete points on ROC curves. In this curve, V40 ≈ 23% (22.75%) represents the cut point with appropriate sensitivity (80.4%) and specificity (80.0%). If V40 is <23% there is a 33.3% (10/30) risk of grade ≥2 AE, which increases to 89.1% (41/46) if V40 is ≥23% (p<0.001).

The predictive ability of V50 to for grade 3 AE was also tested by ROC analysis. The areas under the ROC curve for grade 3 AE were 0.841 (95% CI: 0.730-0.952; p<0.001). In these curves, V50 = 26.5% represents the cut point with sensitivity (80.0%) and specificity (68.9%). If V50 is <26.5% there is a 6.7% (3/45) risk of Grade 3 AE, which increases to 38.7% (12/31) if V50 is ≥26.5% (p = 0.001).

### Occurring time of AE

**Figure 1:**
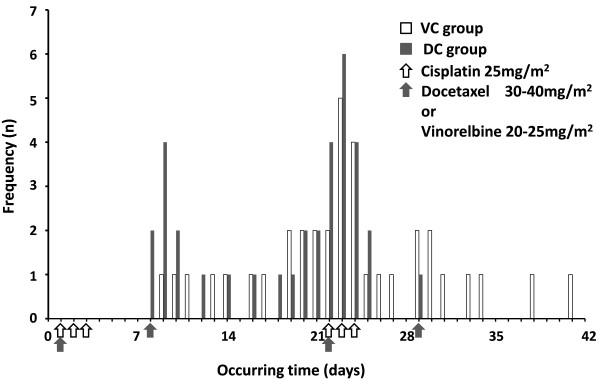
**Distribution of VC and DC group patients according to the occurring time.** Abbreviations: VC = vinorelbine/cisplatin; DC = docetaxel/cisplatin.

All 70 patients experienced grade ≥1 AE were included in the analysis, 36 in VC group, and 34 in DC group. The median occurring time was 22 day (ranging 8-41 day) for all 70 patients, 23 day (ranging 9-41 day) for the VC group (n = 36) and 21.5 day (ranging 8-29 day) for the DC group (n = 34). There were significantly statistical difference between VC and DC groups by Mann–Whitney U Test (Z = -2.465, p = 0.014). Grade ≥1 AE occur later in patients treated with VC than those with DC. Distribution of VC and DC group patients according to the occurring time were depicted in Figure 1.

### Duration of grade ≥1 AE and its predictors

All 70 patients with grade ≥1 AE were included in the duration analysis. Dosimetric parameters of VC and DC groups were summarized in Table 4. There were marginally statistical difference in some dosimetric parameters between VC group and DC group by t Test (p<0.1). Most dosimetric parameters for the VC group were higher than those for the DC group.

**Table 4:** Dosimetric parameter of VC group versus DC group

**Factors**	**VC group (*n*** **= 36)**	**DC group (*n*** **= 34)**	***t***	***p *** **value**
Dmean (Gy)	26.2 ± 9.7	22.6 ± 8.5	1.626	0.108
Dmax (Gy)	67.1 ± 5.3	65.2 ± 9.3	1.074	0.288
V20 (%)	48.2 ± 16.2	41.9 ± 13.2	1.782	0.079
V30 (%)	40.6 ± 16.9	33.7 ± 13.7	1.868	0.066
V40 (%)	34.2 ± 16.4	27.6 ± 15.3	1.727	0.089
V50 (%)	28.3 ± 16.2	21.6 ± 16.1	1.747	0.085
V60 (%)	19.2 ± 14.9	14.2 ± 14.6	1.424	0.159

*Abbreviations*: *VC* vinorelbine/cisplatin, *DC* docetaxel/cisplatin, *Dmax* the maximum dose of the esophagus, *Dmean* the mean dose of the esophagus, V20,30,40,50,60 = relative esophageal volume for radiation dose ≥20,30,40,50,60 Gy, respectively.

The mean ± SD durations of 70 patients suffered grade ≥1 AE were of 41.2 ± 11.1 days. There were significant differences in AE duration between VC group and DC group by t test (t = -2.931, p = 0.005). A longer duration (about one week) was found in DC group (45.0 ± 10.9 days) in comparison to the VC group (37.6 ± 10.3 days).

**Figure 2:**
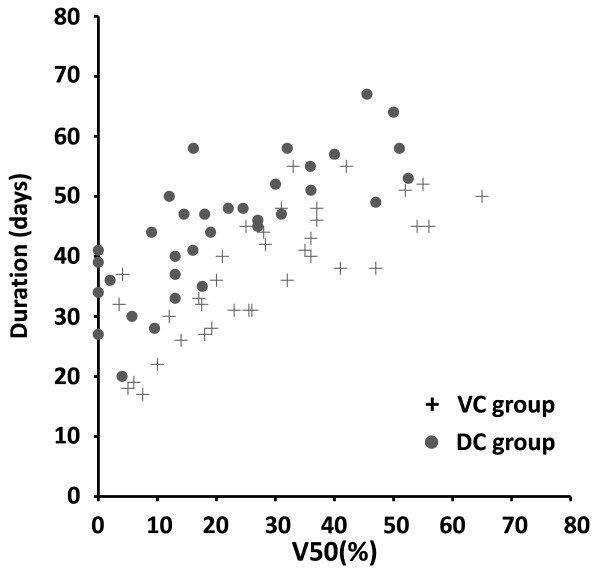
**AE duration versus V50 of esophagus for patients in VC group (+) and DC group (****).** Abbreviations: AE = acute esophagitis; V50 = relative esophageal volume for radiation dose ≥ 50 Gy; VC = Vinorelbine/Cisplatin; DC = Docetaxel/Cisplatin.

By Pearson correlation analysis, CCA (r = 0.335, p = 0.005), V20 (r = 0.466, p < 0.001), V30 (r = 0.593, p < 0.001), V40 (r = 0.628, p < 0.001), V50 (r = 0.650, p < 0.001), V60 (r = 0.599, p < 0.001), Dmax (r = 0.268, p = 0.025) and Dmean (r = 0.630, p < 0.001) were all significantly correlated with grade ≥1 AE duration. Figure 2 shows the plot of the AE duration (days) versus V50 of esophagus for patients with VC group and DC group. On stepwise multiple linear regression analysis, V50 and CCA were significant independent factors affecting grade ≥1 AE duration. Adjusted R^2^ = 0.643. Patients who received concomitant chemotherapy with VC show a shorter duration compared with DC. The predictive equation of AE duration was as follows: Y(days) = 12.195 + 0.512 × V50 (%) + 10.865 × CCA, where V50 (%) = relative esophageal volume for radiation dose ≥50 Gy and CCA = 1 or 2 for concomitant chemotherapy with VC or DC, respectively.

### Discussion

AE is one of the main complications of radiotherapy for NSCLC. Meta-analysis [7] have showed that concomitant chemoradiotherapy significantly increased grade 3 to 4 AE as compared with sequential chemoradiotherapy, from 4% to 18% with a relative risk of 4.9 (95% CI, 3.1 to 7.8; p < 0.001). Severe AE may worsen patients’ quality of life and cause interruption in their treatments. Predicting AE in patients treated with concomitant chemoradiotherapy is essential for clinical treatment planning.

There were few prior reports about the effect of CCA on developing AE. The CALGB study 9431 [8], showed an attractive result about the effect of CCA on developing AE. In the phase II study, vinorelbine group had similar therapeutic efficacy with a lower incidence of Grade ≥3 esophageal toxicity compared with gemcitabine group or paclitaxel group (25% vs. 52% and 39%, respectively). Prior studies on AE involved in a variety of chemotherapy agents and a mixture of sequential, concurrent, or induction plus concomitant treatment with thoracic irradiation. In the present study, we reduced some of these variations. Most of patients had never received chemotherapy, and were administrated concomitant chemotherapy at the same scheduling. Based on the homogeneity, VC group showed lower esophageal toxicity in multivariate analysis, based on not only grade but also duration.

Esophagus is lined by nonkeratinized epithelium with a lamina propria and muscularis mucosa. AE is one of the gastrointestinal mucositis (GIM) caused by cancer therapies. Chemotherapy and radiotherapy damage the dividing and differentiating cells and limit the proliferative ability of the epithelium, so that it becomes thin or ulcerated [9,10]. Chemotherapy also alters the proliferative rate of connective tissue cells within the lamina propria, which results in increased vascular permeability and an inflammatory infiltrate [10]. The plethora of rapidly dividing cells in the gastrointestinal tract makes the tract particularly vulnerable to cytotoxic chemotherapeutic agents [11]. Risk of mucositis has classically been directly associated with modality, intensity and route of delivery of the cancer therapy [12]. Significant changes in the agents or doses commonly employed would be expected to impact the incidence of mucositis [13]. Certain agents, such as 5-fluorouracil, capecitabine, tegafur, methotrexate, irinotecan and the taxanes, lead to a high rate of GIM [11,12,14]. As for the esophagus, many patients undergoing chemotherapy with agents such as methotrexate, 5-fluorouracil, or the taxanes develop esophagitis [14]. There is little information in the literature regarding esophageal mucositis because most symptoms localized to the esophagus usually are attributed to gastroesophageal reflux disease or to either viral or fungal infections, which can coexist with any direct chemotherapy-induced toxicity [10]. At present, there are a limited number of instruments available for assessment of GIM. These scales typically measure indirect outcomes of mucosal injury, including diarrhea [12].

Several studies reported that docetaxel showed heavy GIM compared with vinorelbine in NSCLC patients treated with chemotherapy. In a randomized phase II study of docetaxel or vinorelbine in combination with cisplatin against NSCLC [15], more patients suffered from diarrhea in the DC arm than in the VC arm (45.7% grade 1 to 4 vs. 6.3% grade 1 to 2, p < 0.001). Another phase III study [16] showed that grade 3 to 4 diarrhea occurred more frequently in DC (6.7%) and docetaxel/carboplatin (5.2%) patients compared with VC patients (2.8%), and they had a negligible influence on treatment delays and treatment discontinuation. In the epidemiological study of treatment-associated mucosal injury using modified meta-analysis methods, VC regimen has the lowest incidence of grade 3 or 4 diarrhea (0.25%) compared with other combined regimens using in NSCLC [13]. Vinorelbine maybe show gentle GIM within effective anti-tumor dose range compared with docetaxel.

In the QUANTEC study, Werner-Wasik et al. reviewed many studies on analyzing dosimetric risk factors of AE, the results were not consistent [17]. But there was a clear trend demonstrating that volumes receiving >40-50 Gy correlated significantly with AE [17]. In this study, we found that V40 and V50 were important predictors of AE. Kwint et al. [18] reported that the V50 was identified as the most accurate predictor of grade ≥3 AE for NSCLC patients treated with IMRT and concurrent chemotherapy. In this study, we also found that V50 was a significant independent factor affecting AE duration. This result generally agrees with those reported by Algara M et al. [19] and Rodríguez N et al. [20].

## Conclusions

Patients who had received concomitant chemotherapy with VC show statistically lower AE grade, later occurring time and shorter AE duration compared with the patients treated with DC in the present study. This indicates that the CCA may be a critical influencing factor of AE in these patients. The analysis of subgroup with different CCA will be needed in order to explore the most valuable predictor of AE. Dosimetric parameters V40 and V50 were important predictors of grade ≥2 or ≥3 AE, respectively, for NSCLC patients treated with concomitant chemoradiotherapy.

## Abbreviations

AE: Acute esophagitis; NSCLC: Non-small-cell lung cancer; VC: Vinorelbine/cisplatin; DC: Docetaxel/cisplatin; RTOG: Radiation Therapy Oncology Group; KPS: Karnofsky performance status; CCA: Concomitant chemotherapy agents; V20,30,40,50,60: Percentage of esophagus volume treated to ≥20, ≥30, ≥40, ≥50 and ≥60 Gy; Dmax: The maximum doses delivered to esophagus; Dmean: The mean doses delivered to esophagus; 3D-CRT: Three-dimensional conformal radiotherapy; IMRT: Intensity modulated radiation therapy; CT: Computed Tomography; GTV: Gross tumor volume; PTV: Planning target volume; ABC: Active breathing coordinator; SD: Standard deviation; ROC: Receiver operating characteristic; CALGB: Cancer and leukemia group B; GIM: Gastrointestinal mucositis.

## Competing interests

The authors declare that they have no competing interests.

## Authors’ contributions

ZCZ and JX carried out the manuscript writing; ZCZ, YY and HSL participated in statistical analysis; ZCZ, JX, TZ, HFS, WH and DQW helped to collect data, BSL and GGY conceived and designed this study. All authors read and approved the final manuscript.
